# Evaluation of Potential Effects of CYP3A Inhibition and CYP3A Induction on the Pharmacokinetics of Fruquintinib in Healthy Subjects

**DOI:** 10.1002/cpdd.1520

**Published:** 2025-05-14

**Authors:** Martha Gonzalez, Zhao Yang, William R Schelman, Xiaofei Zhou, Neeraj Gupta, Caly Chien

**Affiliations:** ^1^ HUTCHMED International Corporation Florham Park NJ USA; ^2^ Takeda Development Center Americas, Inc. (TDCA) Cambridge MA USA

**Keywords:** CYP3A, drug‐drug interaction, fruquintinib, itraconazole, rifampin

## Abstract

Cytochrome P450 (CYP) 3A plays a significant role in fruquintinib metabolism in vitro. This 2‐part, 2‐period fixed‐sequence study investigated the impact of CYP3A inhibition (itraconazole) and CYP3A induction (rifampin) on the pharmacokinetics (PK) of fruquintinib and M11, its main metabolite. Fourteen healthy subjects in each part received a single dose of fruquintinib 5 mg alone in Period 1 and with itraconazole (Part A) or rifampin (Part B) in Period 2 under fasted conditions. Itraconazole or rifampin was administered daily 4 or 7 days before coadministration, respectively; administration of both continued throughout the PK sampling period. PK samples were collected before dosing and over 168 hours after fruquintinib dosing. Coadministration with itraconazole resulted in an increase of fruquintinib systemic exposure, determined by area under the plasma concentration‐time curves (AUCs) by approximately 10%. Decreases in M11 AUCs and maximum plasma concentration (C_max_) ranged from 44% to 55% but were not considered clinically meaningful. Rifampin reduced fruquintinib C_max_ and AUCs by 12% and 65%, respectively. Rifampin had a marginal effect on M11 AUCs and increased M11 C_max_ by 2.3‐fold. Data support that concomitant use of fruquintinib with potent CYP3A inducers of rifampin‐like potency should be avoided, but no dose adjustment is recommended when coadministered with CYP3A inhibitors.

Fruquintinib (HMPL‐013) is a highly selective, oral, small‐molecule tyrosine kinase inhibitor of all 3 vascular endothelial growth factor receptors (VEGFR) ‐1, ‐2, and ‐3 that is being developed for the treatment of solid tumors.^1^

Vascular endothelial growth factor (VEGF) is one of the major growth factors related to tumor angiogenesis. It is secreted by tumors and binds to receptors on the surface of endothelial cells to activate the VEGFR signaling pathway, leading to endothelial cell proliferation. Blocking of the VEGF/VEGFR signaling pathway inhibits tumor angiogenesis by cutting off the supply of nutrients and oxygen to the tumor.^2^ The efficacy and safety of fruquintinib was investigated in the Phase 3, multicenter, randomized, double‐blind, placebo‐controlled FRESCO study (NCT02314819), conducted in China. This study met its primary end point, demonstrating significantly improved overall survival with fruquintinib compared with placebo in patients with metastatic colorectal cancer (mCRC) in a third‐line or later setting.^3^ Based on these data, fruquintinib was approved in China, for the treatment of mCRC in patients who had failed 2 prior lines of systemic therapy.^4^ FRESCO‐2 (NCT04322539) was a global, randomized, double‐blind, placebo‐controlled, Phase 3 study, conducted to investigate the efficacy and safety of fruquintinib in patients with refractory mCRC in a third‐line or later setting.^5^ FRESCO‐2 also met its primary end point, demonstrating a significant and clinically meaningful benefit in overall survival with fruquintinib, compared with placebo. Based on the results from FRESCO and FRESCO‐2, fruquintinib was approved in the United States in 2023 for the treatment of adult patients with mCRC who have been previously treated with fluoropyrimidine‐, oxaliplatin‐, and irinotecan‐based chemotherapy, an anti‐VEGF therapy, and, if *RAS* wild‐type and medically appropriate, an antiepidermal growth factor receptor therapy, at a dose of 5 mg once daily 3 weeks on/1 week off in a 28‐day cycle.

Fruquintinib is extensively metabolized in humans into numerous metabolites by multiple enzymes, including cytochrome P450 (CYP [CYP3A and CYP2C subfamilies]) and non‐CYP450 enzyme systems. The proposed primary metabolic pathway is shown in Figure 1. A mass balance study indicated that the metabolic pathways for fruquintinib included oxidation, demethylation, hydrolysis, or further combination with glucuronidation and sulfation, with metabolite M11 (N‐demethylation on the benzofuran ring) being the major circulating metabolite, accounting for approximately 17% of the total radioactivity exposure in plasma.^6^ The potency of M11 for inhibiting VEGFR‐2 kinase activity is 10 times lower than that of fruquintinib. Multiple CYP isoforms were involved in the metabolism of fruquintinib in vitro, and CYP3A had the highest contribution relative to other isoforms.^7^ As such, medications that inhibit or induce CYP3A activity may potentially influence fruquintinib pharmacokinetics (PK) and subsequently its efficacy and safety profiles. This study (NCT04557397) was designed to evaluate the PK characteristics and safety of a single dose of fruquintinib when given alone or coadministered with either itraconazole, a CYP3A inhibitor,^8^ or rifampin, a CYP3A inducer.^9^

**Figure 1:**
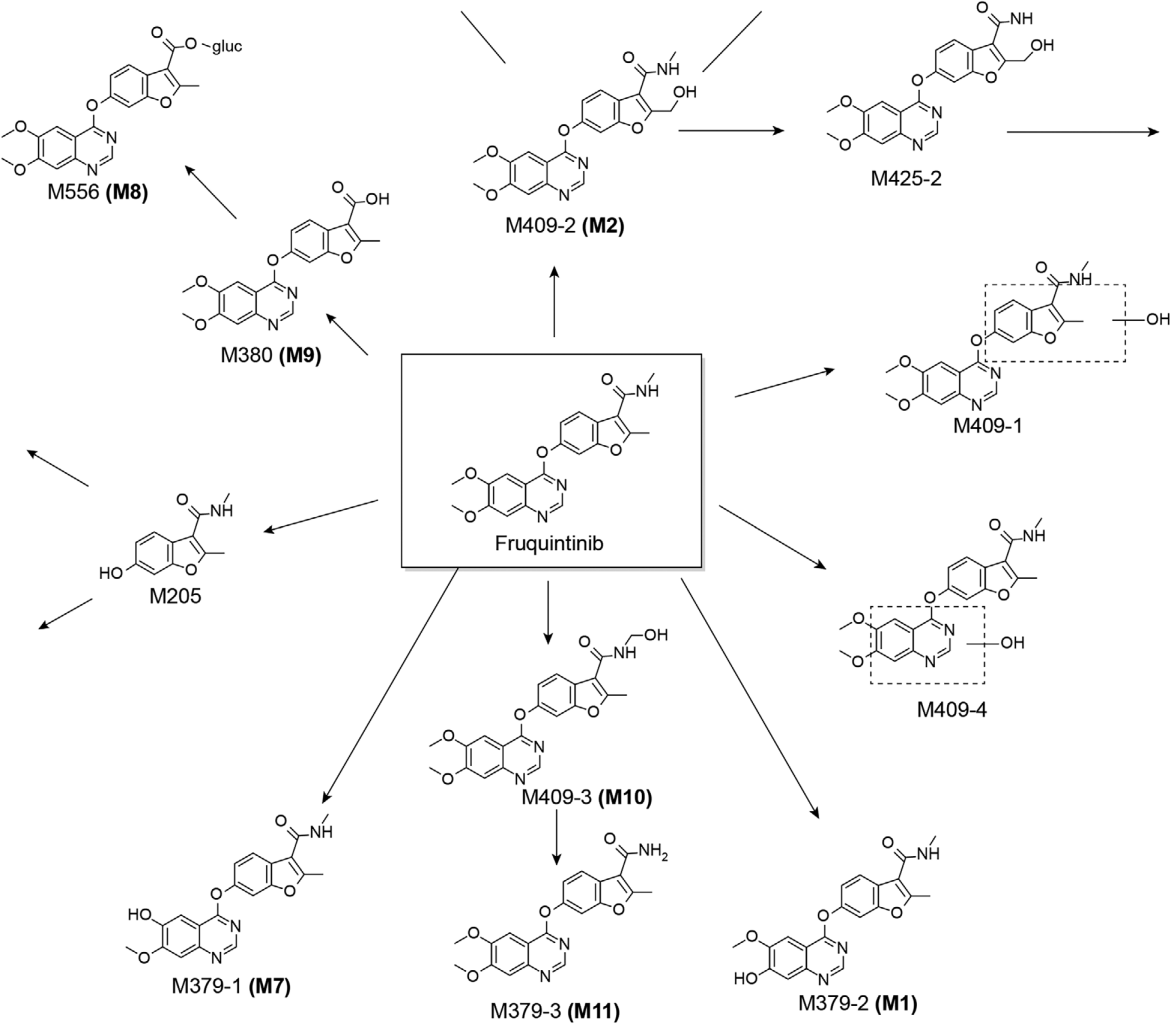
Proposed metabolic pathway for fruquintinib.

## Subjects and Methods

### Study Design

This was a single‐center, open‐label, 2‐part (Part A and Part B), 2‐period, fixed‐sequence study conducted in healthy male and female subjects. Parts A and B evaluated the effect of the CYP3A inhibitor itraconazole and the CYP3A inducer rifampin on the PK profile of fruquintinib, respectively. Each part consisted of a screening phase, a treatment phase (Period 1 and Period 2), and an end‐of‐study (EOS) phase. Screening occurred within 21 days before the first study drug administration. There was at least a 14‐day washout of fruquintinib between treatment periods. PK samples were collected before dosing and over 168 hours following the fruquintinib dose in Part A (with itraconazole) and Part B (with rifampin). Part A and Part B were enrolled independently.

In Part A, subjects were administered fruquintinib alone in Treatment Period 1 and in combination with itraconazole in Treatment Period 2. Study Day 1 was considered the start of Treatment Period 1, and Study Day 15 was considered the start of Treatment Period 2. On Study Day 1, subjects received a single dose of fruquintinib 5 mg by mouth under fasted conditions (subjects fasted for at least 10 hours before fruquintinib dosing and 4 hours after), and on Study Day 19 subjects received a second single dose of fruquintinib 5 mg by mouth under fasted conditions, alongside itraconazole. On Study Day 15, subjects began a regimen of itraconazole 200 mg by mouth twice a day, followed by once daily dosing on Study Days 16‐25. A dose of 200 mg was selected, with a loading dose as part of a 5‐day lead‐in (twice daily for 1 day, once daily for 4 days) to achieve appropriate inhibition of CYP3A before dosing with fruquintinib.^10^ Once‐daily dosing of itraconazole for an additional 5 days throughout the fruquintinib PK sampling period was continued to maintain CYP3A inhibition.^11^

As itraconazole oral bioavailability is maximal when the capsules are taken immediately after a full meal,^12^ it was administered under fed conditions except when coadministered with fruquintinib on Study Day 19. Itraconazole doses were administered 30 minutes after the start of breakfast on Study Days 15‐18 and Study Days 20‐25. On Study Day 19, subjects received both treatments under fasted conditions, and itraconazole was administered 1 hour before administration of a single dose of fruquintinib 5 mg by mouth. Subjects were discharged after completion of EOS procedures on Study Day 26 or at early withdrawal.

In Part B, subjects were administered fruquintinib alone in Treatment Period 1 and in combination with rifampin in Treatment Period 2. Study Day 1 was considered as the start of Treatment Period 1, and Study Day 8 was considered as the start of Treatment Period 2. On Study Day 1, a single dose of fruquintinib 5 mg by mouth was administered under fasted conditions, and on Study Day 15, a single dose of fruquintinib 5 mg was administered by mouth under fasted conditions alongside rifampin. Starting on Study Day 8, rifampin 600 mg was dosed by mouth once daily for 14 consecutive days, 7 days before fruquintinib dosing and 6 days after fruquintinib dosing, to ensure that induction was maintained throughout the PK sampling period. Subjects therefore received rifampin from Study Day 8 to Study Day 21. Absorption of rifampin is reduced by approximately 30% when the drug is ingested with food.^13^ Therefore, rifampin was administered at least 1 hour before breakfast when given alone and under fasted conditions when administered with fruquintinib. Breakfast was given at least 1 hour after the rifampin doses on Study Days 8‐14 and Study Days 16‐21. On Study Day 15, subjects received both treatments under fasted conditions, and rifampin was administered 1hour before administration of a single dose of fruquintinib 5 mg by mouth. Subjects were discharged after completion of EOS procedures on Study Day 22 or at early withdrawal.

### Subject Selection

Healthy, nonsmoking men and women between the ages of 18 and 55 years, with a body mass index of 18‐29 kg/m^2^ were enrolled at WCCT Global Inc (Cypress, CA). Women were of non‐childbearing potential (eg, postmenopausal or surgically sterile), and men agreed to use highly effective contraception. Subjects were excluded from the study if they had evidence of clinically significant cardiovascular, gastrointestinal, hepatic, renal, respiratory, endocrine, hematological, neurological, or psychiatric diseases or abnormalities. Other key exclusion criteria included a known history of any gastrointestinal surgery or any condition possibly affecting drug absorption; a history of smoking or use of nicotine‐containing substances within the previous 2 months or a positive cotinine test at screening (Study Days −21 to −2) and check‐in (Study Day −1) for any one of the treatment periods; a history of drug or alcohol misuse within 6 months before screening or a positive urine drug test at screening or check‐in for any one of the treatment periods; diagnosis of acquired immune deficiency syndrome or positive test for human immunodeficiency virus, hepatitis B virus, or hepatitis C virus; and participation in a clinical study of another drug before the screening, when the time since the last use of the other study drug was less than 5 times the half‐life or 4 weeks, whichever was longer, or the subject was currently enrolled in another clinical study. Consumption of grapefruit; starfruit; Seville oranges; herbal preparations/medications including, but not limited to, kava, ephedra, and *Ginkgo biloba*; dehydroepiandrosterone; yohimbe; saw palmetto; and ginseng within 7 days before the first dose was not allowed.

The protocol and consent form were approved by an institutional review board (Salus Independent Review Board, Austin, TX) before study initiation, and all subjects signed informed consent forms before any study procedures. The study was performed in accordance with the requirements of the Declaration of Helsinki, the International Council for Harmonization Guideline for Good Clinical Practice, and other applicable local laws and regulations.

### Pharmacokinetic Assessments

Blood samples for determination of fruquintinib and metabolite M11 plasma concentrations were collected before dosing and at 0.5, 1, 1.5, 2, 3, 4, 6, 8, 12, 24, 36, 48, 72, 96, 120, 144, and 168 hours after fruquintinib dosing. Plasma samples were analyzed by LabCorp Bioanalytical (Indianapolis, IN) using a validated liquid chromatography with tandem mass spectrometry assay method with an analytical range of 1.00 ng/mL (lower limit of quantitation) to 750 ng/mL (upper limit of quantitation) for both fruquintinib and M11.^6^ Interrun variability was 6.3% or less and 13.8% or less for fruquintinib and M11, respectively. Quality control samples at 4 concentrations (3, 30, 300, and 600 ng/mL) were assayed along with study samples, and the bias quality control sample concentrations deviated by ±2.0% and ±2.7% from the nominal concentrations for fruquintinib and M11, respectively.

PK parameters were determined by noncompartmental analysis and included maximum plasma concentration (C_max_); time to reach C_max_ (t_max_); area under the plasma concentration‐time curve (AUC) from time 0 to the time of the last measurable concentration (AUC_0‐t_); AUC from time 0 to infinity (AUC_0‐inf_); half‐life (t_1/2_); and the metabolite‐to‐parent ratios for C_max_, AUC_0‐t_, and AUC_0‐inf_. Apparent oral clearance and volume of distribution were also estimated for fruquintinib.

The PK of itraconazole (and its metabolite) and rifampin in the regimens used in this study have been extensively investigated.^12^, ^13^, ^14^ Therefore, plasma concentrations of itraconazole and rifampin were not measured in this study.

### Safety Assessments

Safety was assessed by evaluation of adverse events (AEs), serious AEs, AEs of special interest, physical examinations, vital signs, single 12‐lead electrocardiograms and cardiac monitoring, and clinical laboratory data. All AEs were coded using the Medical Dictionary for Regulatory Activities Version 23.0, and their severity was graded using the National Cancer Institute Common Terminology Criteria for Adverse Events Version 5.0.

### Statistical Analysis

Historical data suggest that the maximum intrasubject coefficient of variation is around 15.5% for PK parameters such as C_max_ after administration of a single dose of fruquintinib 5 mg. Assuming a true ratio of geometric mean between test and reference of 1.0, a total of 12 subjects were estimated to give at least 82% power for the 90% confidence interval (CI) for the geometric mean ratio (GMR) falling within the limits of 80% and 125%. Approximately 14 subjects were needed to accommodate a 10% dropout rate in each part of the study.

The PK evaluable population included all subjects who received at least 1 dose of the study drug and had a sufficient PK profile to derive at least 1 PK parameter. For Part A, the effect of itraconazole based on the PK parameters of fruquintinib was evaluated using a linear mixed‐effect model with treatment (ie, fruquintinib with itraconazole [test] and fruquintinib alone [reference]) as fixed effects and subject as a random effect. A similar inferential analysis was performed for Part B, but fruquintinib with rifampin was the test group. For both analyses, the ratios of geometric least square means and their 2‐sided 90% CIs between test and reference were calculated by back transformation (exponent) of least square means from the aforementioned models. PK parameter analyses were based on the PK‐evaluable population. Safety evaluations were performed in the safety population, which included all subjects who received at least 1 dose of fruquintinib.

All PK analyses were conducted using Phoenix^®^ WinNonlin^®^ Version 8.2 or higher (Certara, L.P., Princeton, NJ). All statistical analyses were performed using SAS Version 9.4 or higher (SAS Institute, Cary, NC).

## Results

### Subject Disposition

A total of 28 subjects were enrolled in the study (14 subjects in each part). Subject demographics and baseline characteristics are presented in Table S1. All patients in both parts received at least 1 dose of the study drug, had at least 1 measurable plasma concentration data point and had a sufficient PK profile to derive at least 1 PK parameter, and were included in the safety population, the PK population, and the PK‐evaluable population.

### Effect of Itraconazole on the PK of Fruquintinib and M11

**Figure 2:**
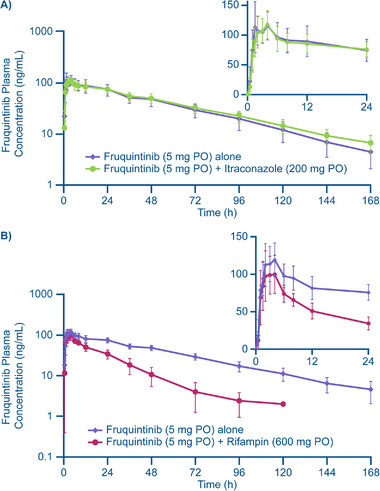
Mean (±SD) Plasma concentration‐time profiles for fruquintinib when given alone or with itraconazole (A) or with rifampin (B). PO, by mouth; SD, standard deviation.

Mean concentration‐time profiles of fruquintinib alone and with itraconazole were comparable (Figure 2A). Mean fruquintinib concentrations peaked rapidly for both groups, followed by a linear decline. The median t_max_ of fruquintinib was delayed 2 hours following coadministration of itraconazole, while the arithmetic mean t_1/2_ increased from approximately 34 hours for fruquintinib alone to approximately 41 hours for fruquintinib with itraconazole (Table 1). Coadministration of itraconazole did not have any significant effect on the systemic exposure of fruquintinib, based on C_max_ and AUCs, as the least squares GMRs of fruquintinib with itraconazole versus fruquintinib alone were between 0.94 and 1.10, and the 90% CIs were within 80%‐125% (Table 1).

**Table 1:** Fruquintinib PK Parameters When Fruquintinib Was Given Alone, With Itraconazole, or With Rifampin

	Part A			Part B		
	Fruquintinib alone (5 mg)	Fruquintinib (5 mg) + itraconazole (200 mg)		Fruquintinib alone (5 mg)	Fruquintinib (5 mg) + rifampin (600 mg)	
PK parameter	(n = 14)	(n = 14)	Least squares GMR (90% CI)	(n = 14^a^)	(n = 13)	Least squares GMR (90% CI)
C_max_ (ng/mL)	133 (36.2)	122 (22.8)	0.94 (0.86–1.03)	123 (25.5)	108 (26.0)	0.88 (0.82–0.94)
AUC_0–t_ (ng•h/mL)	5730 (1390)	6040 (1070)	1.07 (1.01‐1.13)	5640 (789)	2020 (466)	0.35 (0.32–0.40)
AUC_0–inf_ (ng•h/mL)	5960 (1500)	6460 (1240)	1.10 (1.04‐1.16)	5890 (911)	2070 (468)	0.35 (0.31–0.39)
t_max_ (hour)	2.00 (1.50–6.08)	4.00 (1.50–4.20)	–	3.33 (1.50–6.02)	3.00 (1.00–6.00)	–
t_1/2_ (hour)	33.9 (6.96)	41.4 (5.96)	–	33.9 (7.76)	14.0 (3.06)	–
CL/F (mL/min)	14.9 (4.14)	13.3 (2.59)	–	14.5 (2.22)	41.9 (7.46)	–

AUCs, C_max_, t_1/2_, and CL/F are presented as arithmetic mean (SD). t_max_ is presented as median (minimum‐maximum). AUC_0‐inf_, area under the plasma concentration‐time curve from time 0 to infinity; AUC_0‐t_, area under the plasma concentration‐time curve from time 0 to the time of the last measurable concentration; CI, confidence interval; CL/F, apparent oral clearance; C_max_, maximum plasma concentration; GMR, geometric mean ratio; PK, pharmacokinetics; SD, standard deviation.

a n = 13 for AUC_0‐t_, AUC_0‐inf_, t_1/2_, and CL/F.

**Figure 3:**
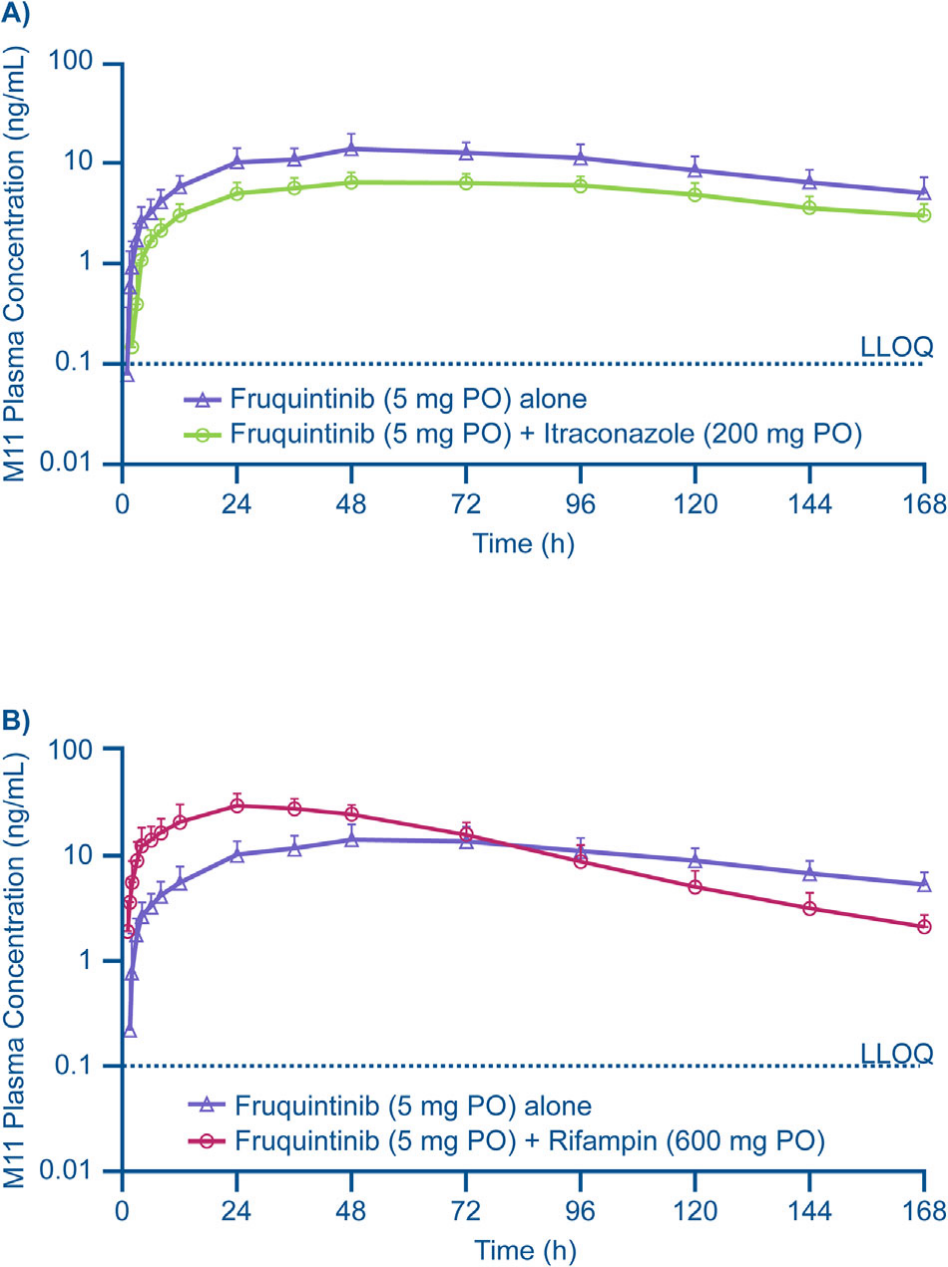
Mean (±SD) plasma metabolite M11 concentrations versus time when fruquintinib was given alone or with itraconazole (A) or with rifampin (B). LLOQ, lower limit of quantitation; PO, by mouth; SD, standard deviation.

The mean concentration‐time profiles of metabolite M11 after administration of fruquintinib alone or with itraconazole are shown in Figure 3A. The rate of formation and overall exposure to metabolite M11 decreased with coadministration of itraconazole: C_max_ decreased by 55%, AUC_0‐t_ by 48%, and AUC_0‐inf_ by 44% (Table 2).

**Table 2:** Metabolite M11 PK Parameters When Fruquintinib Was Given Alone, With Itraconazole, or With Rifampin

	Part A			Part B		
	Fruquintinib alone (5 mg)	Fruquintinib (5 mg) + itraconazole (200 mg)		Fruquintinib alone (5 mg)	Fruquintinib (5 mg) + rifampin (600 mg)	
PK parameter	(n = 14^a^)	(n = 14^b^)	Least squares GMR [90% CI]	(n = 14^c^)	(n = 13)	Least squares GMR (90% CI)
C_max_ (ng/mL)	15.2 (5.61)	6.70 (1.71)	0.45 (0.41–0.49)	13.7 (5.95)	31.1 (7.67)	2.30 (2.02–2.62)
AUC_0‐t_ (ng•h/mL)	1580 (467)	816 (204)	0.52 (0.49–0.56)	1610 (546)	2130 (542)	1.36 (1.17–1.57)
AUC_0‐inf_ (ng•h/mL)	2000 (578)^a^	1100 (295)^b^	0.56 (0.52–0.62)	2160 (666)	2200 (557)	1.04 (0.92–1.17)
t_max_ (hour)	72.0 (48.0–96.0)	47.9 (36.0–120)	–	48.0 (24.0–72.0)	22.9 (22.9–46.9)	–
t_1/2_ (hour)	58.6 (16.6)	70.5 (14.0)	–	71.8 (17.8)	25.1 (5.98)	–
MPR C_max_	0.121 (0.0358)	0.0579 (0.0155)	–	0.117 (0.0462)	0.312 (0.0975)	–
MPR AUC_0‐t_	0.291 (0.0745)	0.141 (0.0301)	–	0.295 (0.0848)	1.12 (0.266)	–
MPR AUC_0‐inf_	0.336 (0.0766)	0.177 (0.0371)	–	0.379 (0.0944)	1.13 (0.269)	–

All PK parameters are presented as t_1/2_ arithmetic mean (SD), except for t_max_ which is presented as median (minimum‐maximum). AUC_0‐inf_, area under the plasma concentration‐time curve from time 0 to infinity; AUC_0‐t_, area under the plasma concentration‐time curve from time 0 to the time of the last measurable concentration; CI, confidence interval; C_max_, maximum plasma concentration; GMR, geometric mean ratio; MPR, metabolite‐to‐parent ratio; PK, pharmacokinetics; SD, standard deviation.

a n = 12 for AUC_0‐inf_, t_1/2_, MPR AUC_0‐inf_.

b n = 13 for AUC_0‐inf_, t_1/2_, MPR AUC_0‐inf_.

c n = 13 for AUC_0‐t_, AUC_0‐inf_, t_1/2_, MPR AUC_0‐t_, MPR AUC_0‐inf_.

### Effect of Rifampin on the PK of Fruquintinib and M11

Mean concentration‐time profiles for fruquintinib with rifampin were lower compared with fruquintinib given alone (Figure 2B). Coadministration of rifampin had minimal effect on the absorption phase of fruquintinib. The geometric mean C_max_ decreased by approximately 12%, and median t_max_ values of fruquintinib were similar for both treatments (3.33 hours for fruquintinib alone compared with 3.00 hours for fruquintinib given with rifampin; Table 1). However, coadministration of rifampin reduced both AUC_0‐t_ and AUC_0‐inf_ by 65% compared with fruquintinib alone, with the 90% CIs around least squares GMRs all below 80%. There was also a marked decrease in the arithmetic mean t_1/2_ of fruquintinib from approximately 34 hours for fruquintinib administered alone to approximately 14 hours for fruquintinib coadministered with rifampin (Table 1).

The increased clearance of fruquintinib when given with rifampin resulted in metabolite M11 t_max_ being reached much earlier, at 23 hours for fruquintinib with rifampin compared with 48 hours for fruquintinib alone (Table 2), and a higher rate of formation and overall exposure to metabolite M11 (Figure 3B). Results from the statistical analyses show that the increase in metabolite M11 exposure with the coadministration of rifampin was greatest for C_max_, with a 2.3‐fold increase, while AUC_0‐t_ increased by 36% with rifampin versus fruquintinib alone. AUC_0‐inf_ was similar between treatments, with the 90% CIs for the least squares GMRs of AUC_0‐inf_ within 80%‐125% (Table 2).

### Safety

There were no AEs reported in Part A (fruquintinib alone or with itraconazole). In Part B (fruquintinib alone or with rifampin), 1 treatment‐emergent AE (back pain) in a single subject (1/14 subjects; 7.1%) was reported after the subject received rifampin alone (Treatment Period 2) and was considered mild (Grade 1) in severity and unrelated to the study treatment.

There were no clinically relevant laboratory test results (serum chemistry, hematology, urinalysis, and coagulation) found during the study. Similarly, there were no clinically relevant findings from assessments of vital signs, 12‐lead electrocardiogram, or physical examination during the study.

## Discussion

This study investigated the impact of itraconazole, a CYP3A inhibitor, and rifampin, a CYP3A inducer, on the fruquintinib PK profile. Understanding these interactions is crucial for optimizing fruquintinib dosing in the clinical setting.

Itraconazole is a potent CYP3A inhibitor and a P‐glycoprotein (P‐gp) inhibitor and is recommended for clinical drug interaction studies.^11^ Ketoconazole has been used as an index CYP3A inhibitor in drug‐drug interaction studies in healthy volunteers. Itraconazole is replacing ketoconazole as an index CYP3A inhibitor recommended by health authorities due to risks of ketoconazole‐associated liver injuries, although research findings do not suggest significant liver toxicities by ketoconazole in drug‐drug interaction studies.^15^, ^16^, ^17^ In addition, a body of evidence suggested that ritonavir or cobicistat may emerge as a more suitable CYP3A index inhibitor than itraconazole because of their CYP3A inhibition potency and safety profiles.^18^, ^19^, ^20^, ^21^ As such, the observed effect size of CYP3A inhibition by itraconazole may be smaller than ritonavir or cobicistat. Fruquintinib is not a P‐gp substrate, so any effect would be due to the CYP3A inhibition. In this study, the inhibition of CYP3A by itraconazole had no clinically significant effect on fruquintinib C_max_, AUC_0‐t_, and AUC_0‐inf_. The least‐squares GMRs of fruquintinib with itraconazole versus fruquintinib alone were close to 1.0, and the 90% CIs were within the 80%–125% limits. However, a decrease in exposure ranging from approximately 45% to 55% for the M11 metabolite of fruquintinib was observed following coadministration with itraconazole, which was statistically significant, with the 90% CIs around the least squares GMRs all being below 1.00. The lack of change in fruquintinib exposure when coadministered with itraconazole indicates that fruquintinib is not a sensitive substrate of CYP3A and its clearance is not solely dependent on CYP3A. This finding is consistent with data from the human mass balance study, which demonstrated that fruquintinib can be metabolized by multiple metabolic pathways in parallel with each other.^6^ These pathways involved CYP enzymes other than CYP3A such as CYP2C8, CYP2C9, and CYP2C19, albeit to a lesser extent, as well as non‐CYP450 enzymes. In the human mass balance study, a substantial amount of metabolite M285 (O‐dequinazoline moiety) and metabolite M381 (O‐dequinazoline moiety) as products of metabolite M205 via sulfation and glucuronidation, respectively, were detected in urine and represented approximately 30% of the administered dose.^6^

The results of this study suggest that these parallel metabolic pathways function as a compensatory mechanism when 1 pathway is inhibited to offset the effect of concomitant medications. The effect of itraconazole on M11 exposure confirmed the in vitro findings that CYP3A is the primary CYP450 enzyme involved in the formation of M11. In vitro, M11 exhibited inhibitory activity against VEGFR2 kinase but the potency was significantly less than that of fruquintinib. Considering that M11 exposure, based on AUCs, and potency is much lower than fruquintinib, the effect of itraconazole on M11 exposure is not considered to be clinically meaningful. Overall, the results suggest that no dose adjustment for fruquintinib is necessary when coadministered with any CYP3A inhibitor.

Rifampin is a CYP3A and P‐gp inducer that is readily absorbed from the gastrointestinal tract.^22^ The induction of CYP3A by rifampin did not have a clinically significant effect on the C_max_ of fruquintinib but reduced the overall exposure of fruquintinib by 65% based on AUCs, in line with an increased activity of CYP3A leading to an increase of metabolite M11 exposure (by 1.36‐fold for AUC_0‐t_ and 2.3‐fold for C_max_). The effects of rifampin on the PK of fruquintinib and M11 are possibly due to several factors. As well as being a inducer of CYP3A, rifampin is also an inducer of CYP enzymes from the 2C family,^23^ all of which are involved in the metabolism of fruquintinib. Therefore, the induction of these CYP enzymes by rifampin resulted in a significant increase in the overall clearance of fruquintinib. The minimal change in AUC of M11 may represent the net effect of increased formation and elimination of M11, as both processes were inducible by rifampin.

Since concomitant use of rifampin resulted in a substantial reduction in fruquintinib systemic exposure, which may reduce fruquintinib efficacy, coadministration of fruquintinib with a CYP3A inducer of rifampin‐like potency should be avoided and alternate concomitant medication with no or minimal CYP3A induction potential considered.

## Conclusion

Coadministration of fruquintinib with itraconazole resulted in the increase of fruquintinib AUCs by approximately 10%, which is not considered clinically meaningful, while coadministration with rifampin significantly decreased fruquintinib exposure. The study results support that no dose adjustment is recommended for fruquintinib when coadministered with CYP3A inhibitors, and that concomitant use of fruquintinib with drugs that are potent CYP3A inducers should be avoided.

## Conflicts of Interest

M.G. was employed by and owned stock in HUTCHMED during the duration of the study and manuscript development. W.R.S. and C.C. are employed by and own stock in HUTCHMED. X.Z. is employed by Takeda Development Center Americas, Inc. N.G. is an employee of and reports ownership of stocks/shares in Takeda. Z.Y. has no conflicts of interest to report.

## Funding

This work was supported by HUTCHMED International Corporation.

## Supporting information



Supporting Information

## Data Availability

The data sets, including the redacted study protocol, redacted statistical analysis plan, and individual participants’ data supporting the results reported in this article, will be made available from the completed study within 3 months of initial request, to researchers who provide a methodologically sound proposal. The data will be provided after its deidentification, in compliance with applicable privacy laws, data protection, and requirements for consent and anonymization.
