# Supplementary material for: Evaluation of Potential Effects of CYP3A Inhibition and CYP3A Induction on the Pharmacokinetics of Fruquintinib in Healthy Subjects
Source: Clin Pharmacol Drug Dev. 2025 May 14;14(8):605–13. doi: 10.1002/cpdd.1520 (PMC12314110; doi:10.1002/cpdd.1520)
Supplement: Supplementary file 1 — Supporting Information [file CPDD-14-605-s001.pdf]

Supplemental Table S1. Subject demographics and baseline characteristics

Characteristic	Part A (*n* = 14)	Part B (*n* = 14)
**Male, n (%)**	9 (64.3)	14 (100)
**Ethnicity, n (%)**		
Hispanic or Latino	6 (42.9)	2 (14.3)
Not Hispanic or Latino	8 (57.1)	12 (85.7)
**Race, n (%)**		
White	11 (78.6)	5 (35.7)
Asian	3 (21.4)	2 (14.3)
Black	0	6 (42.9)
Multiracial	0	1 (7.1)
**Mean age, years (SD)**	42.9 (11.7)	35.6 (7.7)
**Mean weight, kg (SD)**	74.5 (12.9)	78.1 (8.5)
**Mean height, cm (SD)**	169.0 (9.7)	175.2 (7.3)
**Mean BMI, kg/m^2 (SD)**	26.1 (2.6)	25.4 (2.3)

SD= standard deviation
